# Intratumoral heterogeneity score enhances invasiveness prediction in pulmonary ground-glass nodules via stacking ensemble machine learning

**DOI:** 10.1186/s13244-025-02097-0

**Published:** 2025-09-26

**Authors:** Zhichao Zuo, Ying Zeng, Jinqiu Deng, Shanyue Lin, Wanyin Qi, Xiaohong Fan, Yujie Feng

**Affiliations:** 1 Department of Radiology, Xiangtan Central Hospital, 411100 Xiangtan, Hunan Province China; 2 School of Mathematics and Computational Science, Xiangtan University, 411105 Xiangtan, Hunan Province China; 3 Department of Radiology, Affiliated Hospital of Guilin Medical University, 541001 Guilin, The Guangxi Zhuang Autonomous Region China; 4 Department of Radiology, The Affiliated Hospital of Southwest Medical University, 646000 Luzhou, Sichuan Province China; 5 College of Mathematical Medicine, Zhejiang Normal University, 321004 Jinhua, Zhejiang Province China; 6 College of Information and Intelligence, Hunan Agricultural University, 410127 Changsha, Hunan Province China

**Keywords:** Intratumoral heterogeneity score, Stacking classifier, Ternary classification model, Ground-glass nodules, Invasiveness

## Abstract

**Objectives:**

The preoperative differentiation of adenocarcinomas in situ, minimally invasive adenocarcinoma, and invasive adenocarcinoma using computed tomography (CT) is crucial for guiding clinical management decisions. However, accurately classifying ground-glass nodules poses a significant challenge. Incorporating quantitative intratumoral heterogeneity scores may improve the accuracy of this ternary classification.

**Materials and methods:**

In this multicenter retrospective study, we developed ternary classification models by leveraging insights from both base and stacking ensemble machine learning models, incorporating intratumoral heterogeneity scores along with clinical-radiological features to distinguish adenocarcinomas in situ, minimally invasive adenocarcinoma, and invasive adenocarcinoma. The machine learning models were trained, and final model selection depended on maximizing the macro-average area under the curve (macro-AUC) in both the internal and external validation sets.

**Results:**

Data from 802 patients from three centers were divided into a training set (*n* = 477) and an internal test set (*n* = 205), in a 7:3 ratio, with an additional external validation set comprising 120 patients. The stacking classifier exhibited superior performance relative to the other models, achieving macro-AUC values of 0.7850 and 0.7717 for the internal and external validation sets, respectively. Moreover, an interpretability analysis utilizing the Shapley Additive Explanation identified four key features of this ternary classification: intratumoral heterogeneity score, nodule size, nodule type, and age.

**Conclusion:**

The stacking classifier, recognized as the optimal algorithm for integrating the intratumoral heterogeneity score and clinical-radiological features, effectively served as a ternary classification model for assessing the invasiveness of lung adenocarcinoma in chest CT images.

**Critical relevance statement:**

Our stacking classifier integrated intratumoral heterogeneity scores and clinical-radiological features to improve the ternary classification of lung adenocarcinoma invasiveness (adenocarcinomas in situ/minimally invasive adenocarcinoma/invasive adenocarcinoma), aiding in precise diagnosis and clinical decision-making for ground-glass nodules.

**Key Points:**

- The intratumoral heterogeneity score effectively assessed the invasiveness of lung adenocarcinoma.
- The stacking classifier outperformed other methods for this ternary classification task.
- Intratumoral heterogeneity score, nodule size, nodule type, and age predict invasiveness.

**Graphical Abstract:**

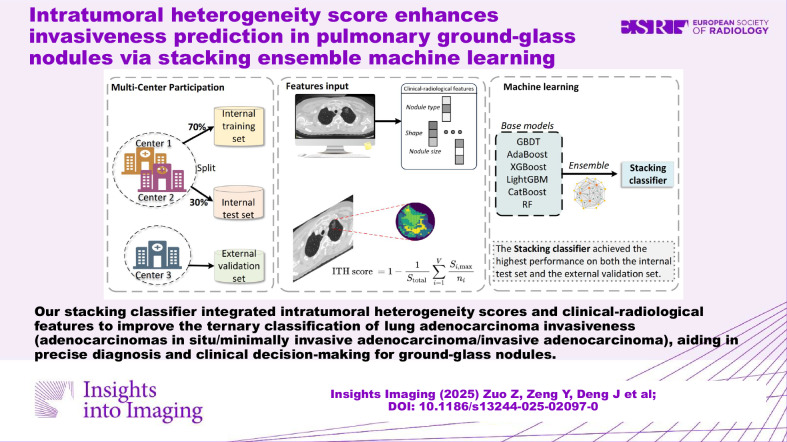

## Introduction

Lung cancer is a leading cause of cancer-related mortality worldwide, with lung adenocarcinoma (LUAD) being the most prevalent histological subtype [1]. Widespread adoption of low-dose computed tomography (CT) screening has significantly increased the detection of early-stage LUADs, which often manifest as ground-glass nodules (GGNs) [2]. Current radiological classifications categorize GGNs as pure ground-glass nodules (pGGNs), which lack solid components, and part-solid nodules (PSNs), containing both ground-glass and solid components [3]. Although pGGNs usually correlate with pre-invasive lesions, such as atypical adenomatous hyperplasia (AAH) or adenocarcinoma in situ (AIS), up to 27% of patients may be diagnosed with invasive adenocarcinoma (IAC) upon pathological examination [4]. Surgical management strategies differ substantially; sublobar resection is often adequate for AAH, AIS, or minimally invasive adenocarcinoma (MIA), whereas IAC typically necessitates lobectomy with lymph node dissection [5–7]. This disparity underscores the need for accurate preoperative classification.

Current challenges in predicting invasiveness primarily stem from overlapping CT features among pGGN subtypes. Traditional diagnostic methods that depend on nodule size or morphological characteristics (e.g., lobulation and spiculation) have demonstrated limited effectiveness [8]. Despite recent advances in computational techniques for lung nodule characterization, these methodologies have significant limitations [9–12]. Although radiomics models are proficient at extracting quantitative imaging features, they often assume uniform tumor heterogeneity, overlooking localized morphological and textural variations that indicate invasive potential [9, 10]. Furthermore, despite their robustness in feature extraction, deep-learning architectures face challenges related to computational inefficiency, dependency on extensively annotated datasets, and a tendency toward overfitting. These issues are compounded by their “black-box” nature, which obscures their mechanistic interpretability and increases their vulnerability to adversarial perturbations [11, 12]. Additionally, the biological and prognostic continuum between AIS, MIA, and IAC necessitates a granular risk stratification approach, as MIA, unlike AIS or IAC, requires distinct therapeutic considerations [13, 14]. However, most studies predominantly use binary classification tasks (e.g., differentiating AAH from IAC [15, 16] or AIS from MIA/IAC [17, 18]), with limited efforts addressing ternary differentiation among AIS, MIA, and IAC [19–21]. Consequently, this oversight creates a significant gap in precision management.

The intratumoral heterogeneity (ITH) score is an innovative quantitative imaging biomarker that provides a robust framework for assessing heterogeneity in pulmonary nodules through the computational analysis of CT-derived pixel patterns [22–25]. This metric is generated through multiscale clustering of label maps. It facilitates dimensionality reduction of high-throughput imaging features while preserving biologically relevant spatial heterogeneity. The ITH score captures the intrinsic tumor complexity associated with the underlying malignant potential by standardizing the interscan variability across heterogeneous datasets. Recent research has indicated that a higher ITH score correlates with greater heterogeneity, establishing it as an effective imaging biomarker that outperforms conventional radiomic approaches for LUADs manifesting as GGNs [22–25].

In this study, we used both base and stacking ensemble machine learning techniques to integrate the ITH score and clinical-radiological features, thereby developing ternary classification models aimed at enhancing the preoperative prediction of invasiveness in LUADs presenting as GGNs [26, 27]. This research has the potential to revolutionize clinical decision-making and provides a reproducible framework for early-stage lung cancer prognosis, addressing critical unmet needs in precision oncology.

## Materials and methods

### Patient enrollment

This multicenter retrospective study was approved by the Institutional Review Boards of Xiangtan Central Hospital (No. 2021-07-009), Affiliated Hospital of Guilin Medical University (No. 2023YJSLL-121), and Affiliated Hospital of Southwest Medical University (No. KY2020147), in compliance with the Declaration of Helsinki. Owing to the retrospective design and anonymization of patient data, informed consent was waived for this study.

Eligible participants were selected based on the following criteria: (1) histopathologically confirmed LUADs presenting as GGNs on preoperative thin-section CT scans (slice thickness ≤ 1.5 mm); (2) nodule diameters measuring 5–30 mm; and (3) no prior biopsy or chemoradiotherapy. The exclusion criteria were as follows: (1) suboptimal quality of imaging or pathological data, (2) a CT scan-to-surgery interval > 6 months, and (3) presence of multifocal primary lung cancers or lung metastatic disease.

Overall, 802 eligible patients from three medical centers were enrolled. Among them, 682 consecutively recruited patients from centers 1 and 2 were randomly divided into training (*n* = 477) and internal validation (*n* = 205) sets, i.e., in a 7:3 ratio. Additionally, 120 prospectively enrolled patients from center 3 formed an independent external validation set. The study enrollment process is shown in Supplementary Fig. 1.

### CT radiological feature evaluation

The CT acquisition protocols in this multicenter study adhered to the standardized parameters established in our previous investigation [24]. All eligible CT images were systematically archived in the picture archiving and communication system for interpretation by three board-certified cardiothoracic radiologists, each with > 10 years of subspecialty experience. They independently evaluated the high-resolution CT images and were blinded to the pathological outcomes. They utilized lung window settings (window level: −600 HU; width: 1500 HU) to optimize the characterization of GGNs.

Several radiological characteristics, including nodule size and type, location, margin, shape, and lobulation, spiculation, vascular convergence, vacuole, and pleural indentation signs, were assessed in accordance with the eighth edition of the Tumor, Node, Metastasis (TNM) classification for lung cancer [28] (Supplementary Material 1).

### ITH score calculation

Following the completion of lung nodule segmentation (Supplementary Material 2), we calculated the ITH score as a quantitative measure to evaluate tumor heterogeneity. This score integrates local pixel features with global pixel distribution patterns derived from the lesion mask. All CT scans in this study were acquired using volumetric protocols with a slice thickness ≤ 1.5 mm, minimizing spatial resolution heterogeneity while maintaining high diagnostic detail.

Referring to previous reports [22–25], this study outlined several critical stages involved in computing the ITH score. First, we applied the ITH score calculation method developed by Li et al [22] for patients with non-small cell lung cancer (https://pypi.org/project/ITHscore), utilizing the “pyradiomics” package and using the “RadiomicsFeatureExtractor()” operator, with all parameters set to their default values for CT image processing. Subsequently, pixel-level feature extraction was performed, wherein a total of 104 pixel-level features (including intensity, texture, and wavelet transforms) were computed for each pixel within the tumor using a 2 × 2-window to capture local pixel characteristics. Following the feature extraction process, the pixels were organized into clusters based on their similarity in features. The optimal number of clusters was established as five [22–25], as this configuration maximizes inter-cluster variance while minimizing intra-cluster variance. Each pixel was then assigned a cluster label, resulting in a clustering label map visually representing distinct tumor subregions. Finally, the ITH score was used as a metric to quantify diversity levels within the label maps, as shown in Eq. (1) below:

1 $${{\rm{I}}}{{\rm{T}}}{{\rm{H}}}\,{{\rm{s}}}{{\rm{c}}}{{\rm{o}}}{{\rm{r}}}{{\rm{e}}}=1-\frac{1}{{S}_{{{\rm{t}}}{{\rm{o}}}{{\rm{t}}}{{\rm{a}}}{{\rm{l}}}}}\sum \limits_{{{\rm{i}}}=1}^{{{\rm{V}}}}\frac{{S}_{1},max}{{n}_{{{\rm{i}}}}}$$

where *V* represents the total number of pixel clusters identified through unsupervised learning (*V* = *5*) and *S*_total_ denotes the aggregate cross-sectional area of the segmented lesion. The parameter *n*_*i*_ indicates the number of topologically distinct regions within cluster *i*, and *S*_*i,*max_ refers to the maximal contiguous area observed in cluster *i*. The ITH scores range from 0 to 1, indicating greater diversity in cell composition and spatial distribution at higher values.

### Ternary classification models based on ensemble stacking classifier

**Fig. 1:**
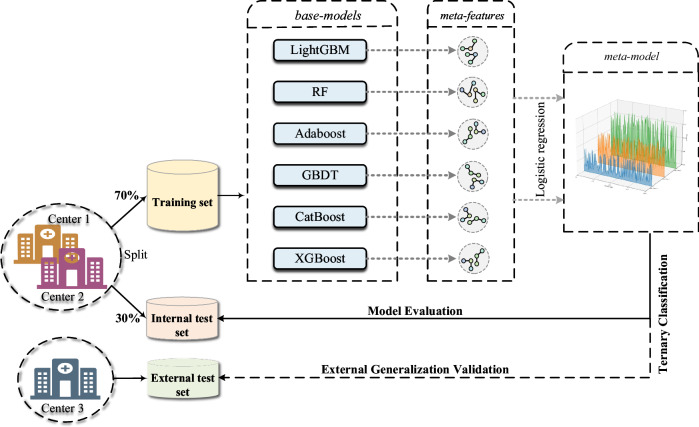
Schematic representation of the stacking classifier architecture

To predict the invasiveness of LUADs manifesting as GGNs, the inputs for the ternary classification model included the ITH score; clinical variables, such as age and sex; and CT radiological features. The detailed framework, illustrated in Fig. 1, presents the ensemble stacking classifier, involving several critical processes. (1) Six supervised machine learning algorithms were used for base model training: random forest, adaptive boosting, light gradient boosting machine, gradient boosting decision tree, extreme gradient boosting, and categorical boosting. These models were trained and fine-tuned on the training set using a five-fold cross-validation approach. Subsequently, a grid search for hyperparameter optimization was performed within these cross-validation loops to ensure robustness and prevent data leakage [26, 27]. (2) After finalizing the base models with their optimal hyperparameters, the predicted values for each machine learning algorithm were calculated to generate meta-features for the second layer of the ensemble learning framework. (3) In the second layer, the meta-model was trained and evaluated using logistic regression on the generated meta-features [29–31]. (4) Following the completion of the ensemble stacking classifier, the external validation set was reserved entirely for the final evaluation, ensuring that performance assessment was unbiased.

### Radiomics analysis

Radiomic analysis follows a structured pipeline comprising three main phases: feature extraction, dimensionality optimization, and predictive modeling [21, 32]. The detailed protocol is provided in Supplementary Material 3.

### Model selection protocol

Base and ensemble machine learning models were developed using the training set. The performance of these models was quantitatively evaluated, with the macro-average receiver operating characteristic (ROC) curve and area under the curve (macro-AUC) serving as the primary evaluation metrics. The macro-AUC metric assesses the performance of each label independently and averages the scores across all labels. Secondary validation metrics included diagnostic accuracy, precision, recall, and F1-score, which were used to assess classification consistency. The final model was selected based on maximizing macro-AUC stability and achieving a value > 0.75 across both internal test and external validation sets [33].

### Model interpretation using the SHapley Additive exPlanation (SHAP) framework

We used SHAP to quantify feature contributions and uncover nonlinear relationships among the ITH scores, clinical-radiological features, and invasiveness predictions in GGNs associated with LUADs to enhance the clinical interpretability of our ensemble models. This approach aligns with the recent methodological advancements in explainable machine learning for medical imaging biomarkers [23, 34]. Details on model interpretation and feature subset identification are provided in Supplementary Material 4.

### Statistical analysis

All statistical analyses and modeling were performed using R (version 3.6.3; R Foundation for Statistical Computing) and Python (version 3.11.0; Python Software Foundation). A detailed protocol is provided in Supplementary Material 5.

## Results

### Patient characteristics

The training set included 477 patients, 90 (18.9%) of whom were diagnosed with AIS; 163 (34.2%), with MIA; and 224 (46.9%), with IAC. The internal validation set comprised 205 patients, of whom 41 (20.0%) were diagnosed with AIS; 65 (31.7%), with MIA; and 99 (48.3%), with IAC. The external validation set comprised 120 patients, of whom 30 (25.0%) were diagnosed with AIS; 38 (31.7%), with MIA; and 52 (43.3%), with IAC.

Detailed characteristics of the enrolled patients are presented in Table 1. No significant differences were detected among the three groups (*p* > 0.05), demonstrating comparability across the groups. Comparative analysis of the clinical-radiological features of AIS, MIA, and IAC within the training set is summarized in Supplementary Table 1.

**Table 1:** Comparative analysis of clinical-radiological characteristics across training, internal test, and external validation sets

Variables	Total (*n* = 802)	Training set (*n* = 477)	Internal test set (*n *= 205)	External validation set (*n* = 120)	*p*-value
Sex, *n* (%)					0.601
Female	559 (69.7)	334 (70)	138 (67.3)	87 (72.5)	
Male	243 (30.3)	143 (30)	67 (32.7)	33 (27.5)	
Age (y), Median (Q1, Q3)	57 (49, 65)	57 (50, 65)	56 (48, 66)	57.5 (49, 66)	0.62
Nodule size (mm), Median (Q1, Q3)	15.3 (10.8, 20.6)	15.8 (11.2, 21.1)	14.4 (10.8, 20)	14.8 (11.1, 19.6)	0.176
Nodule type, *n* (%)					0.562
pGGN	538 (67.1)	314 (65.8)	139 (67.8)	85 (70.8)	
PSN	264 (32.9)	163 (34.2)	66 (32.2)	35 (29.2)	
Location, *n* (%)					0.198
RUL	290 (36.2)	177 (37.1)	75 (36.6)	38 (31.7)	
RML	129 (16.1)	70 (14.7)	33 (16.1)	26 (21.7)	
RLL	52 (6.5)	24 (5)	18 (8.8)	10 (8.3)	
LUL	223 (27.8)	145 (30.4)	48 (23.4)	30 (25)	
LLL	108 (13.5)	61 (12.8)	31 (15.1)	16 (13.3)	
Margin, *n* (%)					0.071
Well-defined	595 (74.2)	340 (71.3)	162 (79)	93 (77.5)	
Ill-defined	207 (25.8)	137 (28.7)	43 (21)	27 (22.5)	
Shape, *n* (%)					0.229
Regular	473 (59)	270 (56.6)	130 (63.4)	73 (60.8)	
Irregular	329 (41)	207 (43.4)	75 (36.6)	47 (39.2)	
Lobulation sign, *n* (%)					0.656
Absent	426 (53.1)	247 (51.8)	113 (55.1)	66 (55)	
Present	376 (46.9)	230 (48.2)	92 (44.9)	54 (45)	
Spiculation sign, *n* (%)					0.262
Absent	501 (62.5)	294 (61.6)	137 (66.8)	70 (58.3)	
Present	301 (37.5)	183 (38.4)	68 (33.2)	50 (41.7)	
Vascular convergence sign, *n* (%)					0.107
Absent	182 (22.7)	96 (20.1)	55 (26.8)	31 (25.8)	
Present	620 (77.3)	381 (79.9)	150 (73.2)	89 (74.2)	
Vacuole sign, *n* (%)					0.412
Absent	661 (82.4)	389 (81.6)	168 (82)	104 (86.7)	
Present	141 (17.6)	88 (18.4)	37 (18)	16 (13.3)	
Pleural indentation sign, *n* (%)					0.082
Absent	399 (49.8)	229 (48)	99 (48.3)	71 (59.2)	
Present	403 (50.2)	248 (52)	106 (51.7)	49 (40.8)	
Label, *n* (%)					0.633
AIS	161 (20)	90 (18.9)	41 (20)	30 (25)	
MIA	266 (33.2)	163 (34.2)	65 (31.7)	38 (31.7)	
IAC	375 (46.8)	224 (46.9)	99 (48.3)	52 (43.3)	

*pGGN* pure ground-glass nodule, *PSN* part-solid nodule, *AIS* adenocarcinoma in situ, *MIA* minimally invasive adenocarcinoma, *IAC* invasive adenocarcinoma, *RUL* right upper lobe, *RLL* right lower lobe, *RML* right middle lobe, *LUL* left upper lobe, *LLL* left lower lobe

### ITH score variability across AIS, MIA, and IAC

**Fig. 2:**
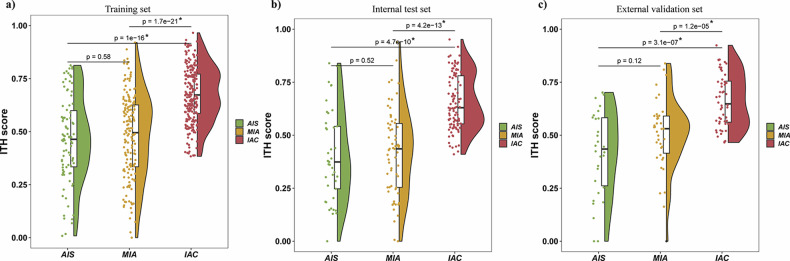
Comparative analysis of intratumoral heterogeneity scores among adenocarcinoma in situ, minimally invasive adenocarcinoma, and invasive adenocarcinoma across the training (**a**), internal test (**b**), and external validation (**c**) sets

Representative visualizations of the ITH scores for the postoperative diagnoses of AIS, MIA, and IAC are shown in Supplementary Fig. 2. Comparison of the ITH scores (Fig. 2a–c) indicated that IAC exhibited a significantly higher ITH score than did both AIS and MIA (*p* < 0.05). MIA demonstrated a tendency toward a higher ITH score than that of AIS but without significance (*p* > 0.05).

### Diagnostic performance

**Fig. 3:**
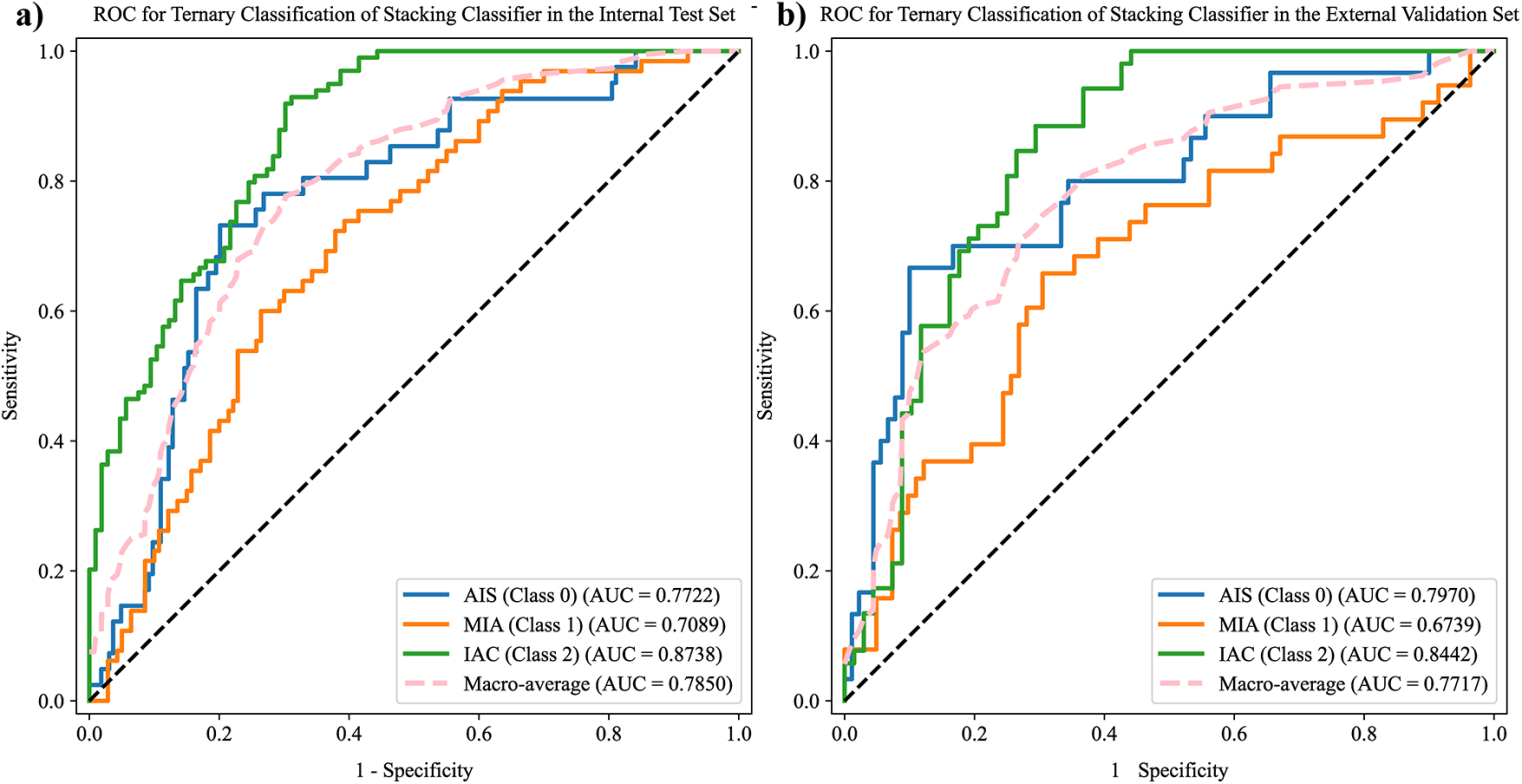
Receiver operating characteristic curve for the stacking classifier’s ternary classification, depicted in the internal test (**a**) and external validation (**b**) sets

We used the ITH score along with clinical-radiological features as input variables for both base and ensemble machine learning models to predict the invasiveness of LUADs presenting as GGNs in our ternary classification task. Table 2 presents the detailed performance metrics for various machine learning models, including the macro-AUC, accuracy, recall, precision, and F1-score. Among these, the stacking classifier achieved the highest macro-AUC, with scores of 0.7850 and 0.7717 in the internal test and external validation sets, respectively, demonstrating its superior overall performance. Figure 3a, b display the ROC curves for the ternary classification of the stacking classifier in both the internal and external validation sets. Additionally, a heat map of the confusion matrices for the ternary classification model (AIS vs. MIA vs. IAC) is shown in Supplementary Fig. 3.

**Table 2:** Assessment of diagnostic efficacy across diverse machine learning approaches

Model	Accuracy	Recall	Precision	F1-score	Macro-AUC
*Internal test set*					
RF	0.6146	0.6146	0.5991	0.5931	0.7785
XGB	0.6000	0.6000	0.5904	0.5925	0.7705
LGBM	0.5805	0.5805	0.5611	0.5685	0.7605
GBM	0.5756	0.5756	0.5585	0.5639	0.7557
AdaBoost	0.5366	0.5366	0.5293	0.5317	0.7320
CatBoost	0.6195	0.6195	0.6000	0.6058	0.7760
Stacking classifier	0.6244	0.6244	0.5146	0.5619	**0.7850**
*External validation set*					
RF	0.5750	0.5750	0.5509	0.5516	0.7616
XGB	0.5667	0.5667	0.5708	0.5547	0.7272
LGBM	0.5500	0.5500	0.5522	0.5395	0.7293
GBM	0.5833	0.5833	0.5750	0.5545	0.7468
AdaBoost	0.5167	0.5167	0.4863	0.4948	0.7076
CatBoost	0.5833	0.5833	0.5775	0.5761	0.7679
Stacking classifier	0.5750	0.5750	0.4458	0.4995	**0.7717**

The bold values indicate the highest Macro-AUC achieved among all machine learning models, both on the Internal Test Set and the External Validation Set. Specifically, the Stacking classifier attained the highest Macro-AUC of 0.7850 internally and 0.7717 externally, highlighting its superior overall diagnostic performance.

*AUC* area under curve, *GBDT* gradient boosting decision tree, *AdaBoost* adaptive boosting, *XGBoost* extreme gradient boosting, *LightGBM* light gradient boosting machine,* CatBoost* categorical boosting, *RF* random forest

**Fig. 4:**
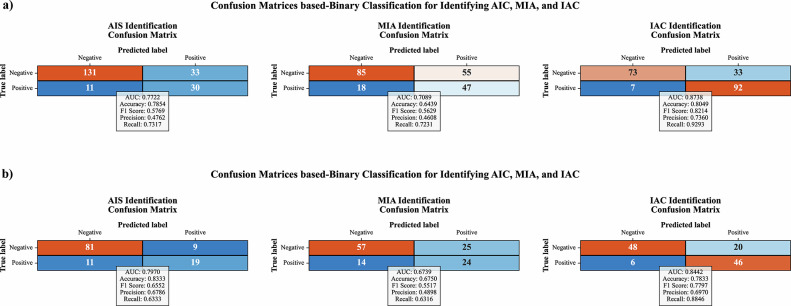
Confusion matrix for the stacking classifier’s binary classification, depicted in the internal test (**a**) and external validation (**b**) sets

Figure 4a, b demonstrate the confusion matrices for the binary classification tasks AIS vs. non-AIS (MIA + IAC), MIA vs. non-MIA (AIS + IAC), and IAC vs. non-IAC (AIS + MIA) in both the internal and external validation sets. Threshold optimization using Youden’s index was performed in these binary classification tasks to enhance performance. The model demonstrated an exceptional capability to predict IAC. It achieved AUC scores of 0.8738 and 0.8442 (strong) for the internal and external validation sets, respectively. The respective AUC scores were 0.7722 and 0.7970 (moderate) for AIS and 0.7089 and 0.6739 (weak) for MIA.

Subgroup analysis revealed notable differences in the diagnostic performance of the stacking classifier between the pGGN and PSN subtypes (Supplementary Figs. 4 and 5). Further details are provided in Supplementary Material 6.

### Model interpretation

**Fig. 5:**
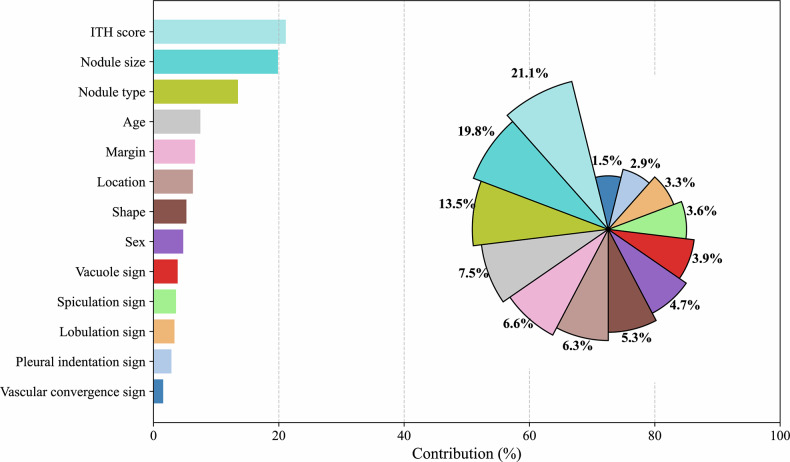
Comprehensive elucidation of the stacking classifier through SHapley Additive exPlanation values, illustrated via a bar graph and a detailed rose plot view

Figure 5 provide a comprehensive interpretation of the stacking classifier using the SHAP values presented through both a bar graph and zoomed-in view of the rose plot. This analysis indicated that the ITH score (21.2%) is the feature making the most substantial contribution to the model.

**Fig. 6:**
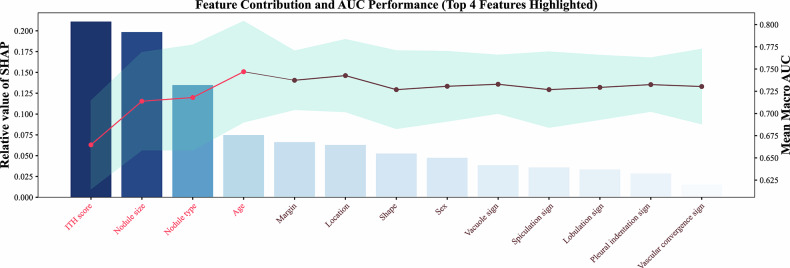
Assessment of the contributions of various features to the model’s predictive performance, accompanied by their respective macro-area under the curve scores

The four most influential features—ITH score, nodule size, nodule type, and age—consistently exhibited significantly higher contribution values than the other variables did in the SHAP-based feature subset identification (Fig. 6). The ITH score demonstrated the greatest contribution, as indicated by a substantial increase in the cumulative macro-AUC value. The other three features also showed a progressive increase in both contribution magnitude and cumulative macro-AUC. However, a gradual decline followed by a plateau in the cumulative macro-AUC was observed with the addition of supplemental model features.

### Comparing the performance of the stacking classifier, radiomics model, and ITH score

The radiomics model (Supplementary Fig. 6) underperformed, compared with both the ITH score and the stacking classifier, with the stacking classifier achieving the best performance (Table 3 and Supplementary Fig. 7). Further details are provided in Supplementary Material 7.

**Table 3:** Comparative evaluation of diagnostic performance among stacking classifier, ITH score, and radiomics model in internal and external validation sets

Model	Accuracy	Recall	Precision	F1-score	Macro-AUC
*Internal test set*					
Stacking classifier	0.6244	0.6244	0.5146	0.5619	0.7850
Radiomics model	0.4829	0.4829	0.4781	0.4372	0.6515
ITH score	0.6098	0.4931	0.4000	0.4416	0.7435
*External validation set*					
Stacking classifier	0.5750	0.5750	0.4458	0.4995	0.7717
Radiomics model	0.4167	0.4167	0.3345	0.3457	0.6161
ITH score	0.5417	0.4615	0.5132	0.4075	0.7116

*AUC* area under curve, *ITH* intratumor heterogeneity

## Discussion

We implemented both base and ensemble machine learning models to integrate clinical-radiological features with the ITH score. The stacking classifier demonstrated the highest predictive performance for the ternary classification model. Additionally, three binary classification models were developed: the stacking classifier exhibited superior performance in predicting IAC, moderate performance in predicting AIS, and the lowest performance in predicting MIA. The ITH score outperformed the traditional radiomics models in ternary classification tasks, whereas the stacking classifier surpassed both methods for the same classification tasks, aligning with the findings of prior research [22–25].

Although radiomics models excel at extracting quantitative imaging features, they often assume uniform tumor heterogeneity, overlooking localized morphological and textural variations that may indicate invasive potential [9, 10]. To address this limitation, an ITH score was developed by clustering label maps at multiple scales, effectively reducing the dimensionality of imaging features while preserving the essential spatial differences. Additionally, it standardized variability across diverse datasets, providing a more accurate representation of the inherent complexity and malignant potential [22–25]. This aligns with this study, in which the radiomics model underperformed, compared with the ITH score.

The stacking classifier integrates features from various base models and utilizes a trainable meta-model, such as logistic regression, to capture nonlinear interactions. This enhances interpretability and robustness by leveraging the strengths of multiple base models while mitigating their weaknesses, thereby providing a solid foundation for the integration of multimodal biomarkers [29–31, 35].

The stacking-classifier-based ternary classification framework was decomposed into three binary models, precisely stratifying the performance levels. Similar to the stacking classifier results, the subgroup analysis of pGGNs revealed superior discriminative capacity in predicting IAC, moderate efficacy in identifying AIS, and diminished effectiveness in classifying MIA, mirroring the biological continuum of tumor aggressiveness. High model confidence correlates strongly with histopathologically confirmed invasiveness in IAC, whereas low-risk predictions align with indolent AIS phenotypes. The model’s diagnostic limitations for MIA stem from inherent biological ambiguity, as intermediate pathological characteristics (lepidic growth with invasion ≤ 5 mm) create an overlapping feature space that complicates binary classification [28]. Therefore, the clinical utility of this system lies primarily in its ability to categorize lesions definitively into low- (AIS) and high-risk (IAC) groups, aiding surgical decision-making.

In this study, the SHAP-based interpretability analysis identified four dominant features with significantly high SHAP values: ITH score, nodule size, nodule type, and age. The ITH score has emerged as the most influential predictor, underscoring its established role in quantifying tumor heterogeneity—a critical biomarker reflecting biological processes, such as dysregulated angiogenesis and heterogeneous cellular proliferation. Nodule size reflects an infiltrative invasive growth pattern according to the eighth edition of the TNM classification for lung cancer [28]. Additionally, nodule type is a surrogate marker for tumor invasiveness in LUAD, especially GGNs, where a higher solid component is linked to aggressive pathological features. This observation aligned with those of previous studies, indicating that PSNs are more invasive than pGGNs [36, 37]. Finally, advanced age has been associated with tumor invasiveness in LUADs presenting as GGNs, corroborating earlier research [36, 37].

This study has some limitations. First, the retrospective multicenter design introduced heterogeneity in CT acquisition protocols due to variations in scanner manufacturers across institutions, affecting texture feature extraction and model generalizability. Second, although the stacked ensemble architecture demonstrated valuable diagnostic utility for preoperatively stratifying GGN pulmonary nodules, its implementation requires segregated training datasets for each hierarchical layer and substantial computational resources for multi-algorithm integration, thereby increasing the computational demands and algorithmic complexity. Third, the exclusive focus on surgically resected specimens introduced spectrum bias, as non-operatively managed cases (which may represent indolent phenotypes) were systematically excluded. Finally, the absence of longitudinal postoperative surveillance data limited the assessment of prognostic model validity regarding clinical outcomes beyond histopathological classifications.

## Conclusion

A stacking-classifier-based ternary classification framework significantly enhanced the preoperative invasive assessment of GGNs by integrating multiple data streams. Incorporating parameters such as ITH score, nodule size, nodule type, and age provides valuable insights into clinical-radiological characteristics and tumor heterogeneity. This study may revolutionize clinical decision-making, as this approach may facilitate the development of personalized management strategies for early-stage lung cancer, addressing critical unmet needs in precision oncology.

## Supplementary information

ELECTRONIC SUPPLEMENTARY MATERIAL

## Data Availability

The data that support the findings of this study are available from the corresponding author upon reasonable request.

## Abbreviations

AIS: Adenocarcinoma in situ
AUC: Area under the curve
CT: Computed tomography
GGN: Ground-glass nodule
ITH: Intratumoral heterogeneity
IAC: Invasive adenocarcinoma
LUAD: Lung adenocarcinoma
MIA: Minimally invasive adenocarcinoma
PSN: Part-solid nodule
pGGN: Pure ground-glass nodule
ROC: Receiver operating characteristic
SHAP: Shapley Additive Explanation

## References

[1] Zhang C, Zhang J, Xu FP et al (2019) Genomic landscape and immune microenvironment features of preinvasive and early invasive lung adenocarcinoma. J Thorac Oncol 14:1912–1923 31446140 10.1016/j.jtho.2019.07.031 PMC6986039

[2] Hattori A, Matsunaga T, Hayashi T, Takamochi K, Oh S, Suzuki K (2017) Prognostic impact of the findings on thin-section computed tomography in patients with subcentimeter non-small cell lung cancer. J Thorac Oncol 12:954–962 28257958 10.1016/j.jtho.2017.02.015

[3] Zuo Z, Zeng W, Peng K et al (2023) Development of a novel combined nomogram integrating deep-learning-assisted CT texture and clinical-radiological features to predict the invasiveness of clinical stage IA part-solid lung adenocarcinoma: a multicentre study. Clin Radiol 78:e698–e706 37487842 10.1016/j.crad.2023.07.002

[4] Woo W, Kang DY, Cha YJ et al (2024) Histopathologic fate of resected pulmonary pure ground glass nodule: a systematic review and meta-analysis. J Thorac Dis 16:924–934 38505083 10.21037/jtd-23-1089 PMC10944737

[5] Saji H, Okada M, Tsuboi M et al (2022) Segmentectomy versus lobectomy in small-sized peripheral non-small-cell lung cancer (JCOG0802/WJOG4607L): a multicentre, open-label, phase 3, randomised, controlled, non-inferiority trial. Lancet 399:1607–1617 35461558 10.1016/S0140-6736(21)02333-3

[6] Dai C, Shen J, Ren Y et al (2016) Choice of surgical procedure for patients with non-small-cell lung cancer ≤ 1 cm or > 1 to 2 cm among lobectomy, segmentectomy, and wedge resection: a population-based study. J Clin Oncol 34:3175–3182 27382092 10.1200/JCO.2015.64.6729

[7] Altorki N, Wang X, Kozono D et al (2023) Lobar or sublobar resection for peripheral stage IA non-small-cell lung cancer. N Engl J Med 388:489–498 36780674 10.1056/NEJMoa2212083 PMC10036605

[8] Hoye J, Solomon J, Sauer TJ, Robins M, Samei E (2019) Systematic analysis of bias and variability of morphologic features for lung lesions in computed tomography. J Med Imaging (Bellingham 6:013504 30944842 10.1117/1.JMI.6.1.013504 PMC6434334

[9] Gillies RJ, Kinahan PE, Hricak H (2016) Radiomics: images are more than pictures, they are data. Radiology 278:563–577 26579733 10.1148/radiol.2015151169 PMC4734157

[10] Zuo Z, Deng J, Ge W et al (2025) Quantifying intratumoral heterogeneity within sub-regions to predict high-grade patterns in clinical stage I solid lung adenocarcinoma. BMC Cancer 25:51 39789523 10.1186/s12885-025-13445-0 PMC11720805

[11] Gong J, Liu J, Hao W et al (2020) A deep residual learning network for predicting lung adenocarcinoma manifesting as ground-glass nodule on CT images. Eur Radiol 30:1847–1855 31811427 10.1007/s00330-019-06533-w

[12] Ni Y, Yang Y, Zheng D, Xie Z, Huang H, Wang W (2020) The invasiveness classification of ground-glass nodules using 3D attention network and HRCT. J Digit Imaging 33:1144–1154 32705434 10.1007/s10278-020-00355-9 PMC7649842

[13] Landreneau RJ, Normolle DP, Christie NA et al (2014) Recurrence and survival outcomes after anatomic segmentectomy versus lobectomy for clinical stage I non-small-cell lung cancer: a propensity-matched analysis. J Clin Oncol 32:2449–2455 24982447 10.1200/JCO.2013.50.8762 PMC4121502

[14] Yotsukura M, Asamura H, Motoi N et al (2021) Long-term prognosis of patients with resected adenocarcinoma in situ and minimally invasive adenocarcinoma of the lung. J Thorac Oncol 16:1312–1320 33915249 10.1016/j.jtho.2021.04.007

[15] Ding H, Xia W, Zhang L et al (2020) CT-based deep learning model for invasiveness classification and micropapillary pattern prediction within lung adenocarcinoma. Front Oncol 10:1186 32775302 10.3389/fonc.2020.01186 PMC7388896

[16] Zuo Z, Wang P, Zeng W, Qi W, Zhang W (2023) Measuring pure ground-glass nodules on computed tomography: assessing agreement between a commercially available deep learning algorithm and radiologists’ readings. Acta Radiol 64:1422–1430 36317301 10.1177/02841851221135406

[17] Xu Y, Li Y, Yin H, Tang W, Fan G (2021) Consecutive serial non-contrast CT scan-based deep learning model facilitates the prediction of tumor invasiveness of ground-glass nodules. Front Oncol 11:725599 34568054 10.3389/fonc.2021.725599 PMC8461974

[18] Wang J, Chen X, Lu H et al (2020) Feature-shared adaptive-boost deep learning for invasiveness classification of pulmonary subsolid nodules in CT images. Med Phys 47:1738–1749 32020649 10.1002/mp.14068

[19] Zhao W, Yang J, Sun Y et al (2018) 3D deep learning from CT scans predicts tumor invasiveness of subcentimeter pulmonary adenocarcinomas. Cancer Res 78:6881–6889 30279243 10.1158/0008-5472.CAN-18-0696

[20] Yanagawa M, Niioka H, Hata A et al (2019) Application of deep learning (3-dimensional convolutional neural network) for the prediction of pathological invasiveness in lung adenocarcinoma: a preliminary study. Medicine (Baltimore) 98:e16119 31232960 10.1097/MD.0000000000016119 PMC6636940

[21] Pan Z, Hu G, Zhu Z et al (2024) Predicting invasiveness of lung adenocarcinoma at chest CT with deep learning ternary classification models. Radiology 311: e232057 38591974 10.1148/radiol.232057

[22] Li J, Qiu Z, Zhang C et al (2023) ITHscore: comprehensive quantification of intra-tumor heterogeneity in NSCLC by multi-scale radiomic features. Eur Radiol 33:893–903 36001124 10.1007/s00330-022-09055-0

[23] Zheng H, Chen W, Qi W, Liu H, Zuo Z (2024) Enhancing the prediction of the invasiveness of pulmonary adenocarcinomas presenting as pure ground-glass nodules: integrating intratumor heterogeneity score with clinical-radiological features via machine learning in a multicenter study. Digit Health 10:20552076241289181 39381817 10.1177/20552076241289181 PMC11459516

[24] Qi H, Zuo Z, Lin S et al (2025) Assessment of intratumor heterogeneity for preoperatively predicting the invasiveness of pulmonary adenocarcinomas manifesting as pure ground-glass nodules. Quant Imaging Med Surg 15:272–286 39839051 10.21037/qims-24-734 PMC11744125

[25] Zhang J, Sha J, Liu W, Zhou Y, Liu H, Zuo Z (2024) Quantification of intratumoral heterogeneity: distinguishing histological subtypes in clinical T1 stage lung adenocarcinoma presenting as pure ground-glass nodules on computed tomography. Acad Radiol 31:4244–4255 38627129 10.1016/j.acra.2024.04.008

[26] Ren Y, Zhang L, Suganthan PN (2016) Ensemble classification and regression - recent developments, applications and future directions. IEEE Comput Intell Mag 11:41–53

[27] Zhang M, Zheng Y, Maidaiti X, Liang B, Wei Y, Sun F (2024) Integrating machine learning into statistical methods in disease risk prediction modeling: a systematic review. Health Data Sci 4:0165 39050273 10.34133/hds.0165 PMC11266123

[28] Minato H, Katayanagi K, Kurumaya H et al (2022) Verification of the eighth edition of the UICC-TNM classification on surgically resected lung adenocarcinoma: Comparison with previous classification in a local center. Cancer Rep 5:e1422 10.1002/cnr2.1422 PMC8789611 34169671

[29] Zhong J, Zeng X, Cao W et al (2022) Semisupervised multiple choice learning for ensemble classification. IEEE Trans Cybern 52:3658–3668 32924945 10.1109/TCYB.2020.3016048

[30] Ling T, Zuo Z, Huang M, Ma J, Wu L (2025) Stacking classifiers based on integrated machine learning model: fusion of CT radiomics and clinical biomarkers to predict lymph node metastasis in locally advanced gastric cancer patients after neoadjuvant chemotherapy. BMC Cancer 25:834 40329193 10.1186/s12885-025-14259-w PMC12057267

[31] Mahajan P, Uddin S, Hajati F, Moni MA (2023) Ensemble learning for disease prediction: a review. Healthcare 11:1808 10.3390/healthcare11121808 PMC10298658 37372925

[32] Karthik KV, Rajalingam A, Shivashankar M, Ganjiwale A (2022) Recursive feature elimination-based biomarker identification for open neural tube defects. Curr Genomics 23:195–206 36777008 10.2174/1389202923666220511162038 PMC9878829

[33] Obuchowski NA, Bullen JA (2018) Receiver operating characteristic (ROC) curves: review of methods with applications in diagnostic medicine. Phys Med Biol 63:07tr01 29512515 10.1088/1361-6560/aab4b1

[34] Azodi CB, Tang J, Shiu SH (2020) Opening the black box: interpretable machine learning for geneticists. Trends Genet 36:442–455 32396837 10.1016/j.tig.2020.03.005

[35] Li Y, Ding J, Wu K et al (2025) Ensemble machine learning classifiers combining CT radiomics and clinical-radiological features for preoperative prediction of pathological invasiveness in lung adenocarcinoma presenting as part-solid nodules: a multicenter retrospective study. Technol Cancer Res Treat 24:15330338251351365 40525253 10.1177/15330338251351365 PMC12174711

[36] Xiong TW, Gan H, Lv FJ, Zhang XC, Fu BJ, Chu ZG (2024) Artificial intelligence-measured nodule mass for determining the invasiveness of neoplastic ground glass nodules. Quant Imaging Med Surg 14:6698–6710 39281163 10.21037/qims-24-665 PMC11400670

[37] Sun JD, Sugarbaker E, Byrne SC et al (2024) Clinical outcomes of resected pure ground-glass, heterogeneous ground-glass, and part-solid pulmonary nodules. AJR Am J Roentgenol 222:e2330504 38323785 10.2214/AJR.23.30504 PMC11161307

