# Supplementary material for: Intratumoral heterogeneity score enhances invasiveness prediction in pulmonary ground-glass nodules via stacking ensemble machine learning
Source: Insights Imaging. 2025 Sep 26;16:209. doi: 10.1186/s13244-025-02097-0 (PMC12474818; doi:10.1186/s13244-025-02097-0)
Supplement: Supplementary file 1 — ELECTRONIC SUPPLEMENTARY MATERIAL [file 13244_2025_2097_MOESM1_ESM.pdf]

Intratumoral Heterogeneity Score Enhances Invasiveness Prediction in Pulmonary Ground-Glass Nodules via Stacking Ensemble Machine Learning

ELECTRONIC SUPPLEMENTARY MATERIAL

Supplementary Material 1

CT Radiological Features

All eligible CT images were systematically archived in the picture archiving and communication system for interpretation by three board-certified cardiothoracic radiologists, each with over 10 years of subspecialty experience. The lesion size of PSN was determined by measuring the maximal axial diameter of the solid component, defined as hyperattenuating parenchymal areas that completely obscure the underlying bronchovascular structures. In contrast, pGGN were measured along their longest transaxial dimension and characterized by increased hazy attenuation while preserving the vascular architecture.

In addition to nodule size and type, several radiological characteristics were evaluated. These included location, margin, shape, lobulation sign, spiculation sign, vascular convergence sign, vacuole sign, and pleural indentation sign.

Nodule location was categorized into five groups: right upper lobe, right middle lobe, right lower lobe, left upper lobe, and left lower lobe. Margins were classified as well-defined or ill-defined, and shape was described as either regular or irregular. The lobulation sign indicates a nodule surface with multiple arcs or undulations resembling lobes. The spiculation sign is characterized by linear strands extending from the nodule margin into the surrounding parenchyma. The vascular convergence sign refers to vessels converging toward the nodule from the adjacent lung tissue. The vacuole sign denotes small intranodular air-filled cavities, typically 1–2 mm in size. Finally, the pleural indentation sign appears as a linear band connecting the nodule to the pleura.

Supplementary Material 2

Nodule Segmentation

Nodule segmentation was performed using ITK-SNAP (v3.6.0, http://www.itksnap.org) for all CT images. The initial manual contouring of the nodule boundaries was performed by a board-certified cardiothoracic radiologist with a decade of experience in thoracic oncology imaging. Subsequently, an attending thoracic radiologist with 15 years of subspecialty experience in thoracic imaging conducted iterative refinements to optimize the segmentation accuracy.

Supplementary Material 3

Radiomic analysis

In the initial phase, we extracted 1,239 quantitative imaging features per lung nodule using Pyradiomics. To standardize these features, we performed normalization through a z-score transformation. This quantitative radiomic profiling of preoperative thin-section CT scans led to the identification of 1,239 candidate radiomic features for each lung lesion.

To predict the invasiveness of LUAD manifesting as GGNs, we employed a rigorous dimensionality reduction protocol. This protocol consisted of a multi-stage pipeline designed to optimize feature selection for our ternary classification radiomics model. We began with a univariate analysis of variance to identify statistically significant features ($p<0.05$), enabling us to eliminate variables that lacked discriminative power. Subsequently, we implemented recursive feature elimination with cross-validation (RFECV) as an iterative refinement step. Initially, the classifier was trained on the complete feature set, followed by the systematic elimination of the least contributory features using 5-fold cross-validation. At each iteration, we evaluated model accuracy until we derived an optimal subset of features. This method leverages RFECV's built-in cross-validation framework to enhance feature relevance while minimizing the risk of overfitting. Finally, we applied L1-regularized (Lasso) regression for sparse feature selection, which penalizes non-informative coefficients by setting them to zero, further refining the feature space.

Supplementary Material 4

SHapley Additive exPlanations (SHAP) Interpretation and Feature Subset Identification

For the stacking classifier, KernelSHAP was employed to accommodate heterogeneous base learners, ensuring model-agnostic interpretability. Additionally, we aggregated all SHAP visualizations across 5-fold cross-validation splits to enhance robustness against sampling variability. The SHAP implementation utilized the “shap” Python package, which performed 100 permutation rounds for estimating background distribution through k-means clustering of the training data.

Furthermore, to identify the most discriminative feature subset, we implemented a dynamic selection protocol that employs SHAP for hierarchical feature prioritization. This approach allows for a dynamic assessment of how modifications in the feature space influence macroAUC trajectories across training iterations. The algorithm operates through sequential model retraining cycles, during which features are incrementally incorporated based on their predictive contributions as determined by SHAP.

The iterative feature elimination process is formalized as an optimization problem. Let F denote the full feature set, and $F_k \subset F$ represent the subset retaining the top k features ranked by SHAP values

(i.e., $F_k = \{f^{(1)}, f^{(2)}, \ldots, f^{(k)}\}$, where $f^{(i)}$ is the *i*-th most important feature). The objective is to identify the smallest $k*$ that balances predictive stability and feature sparsity, defined as follows:

$k*$=min {k∈ {0,1, …, n} =macro*AUC* (F_{k+1}) − macro*AUC* $(F_k) \leq \varepsilon$},

where macro*AUC* (F_k) represents the performance when trained on F_k, and ε is a predefined positive number close to 0. The framework iteratively removes the least important features (starting with k=1), recalculates macro*AUC* (F_k) at each step, and terminates when the condition macro*AUC* (F_{k+1}) − macro*AUC* $(F_k) \leq \varepsilon$ is first satisfied, at which point F_k is selected.

This approach effectively integrates SHAP-based feature ranking, sequential elimination, and *macroAUC*-driven validation into a cohesive optimization process. Notably, after each integration step, model performance is rigorously validated through *macroAUC* recalibration using an internal test set.

Supplementary Material 5

Statistical Analysis

Descriptive statistics were generated using the “tableone” package. The distribution of continuous variables was first assessed for normality: normally distributed variables are reported as mean ± standard deviation, while non-normally distributed variables are summarized as median and interquartile range. Categorical variables were presented as frequencies and percentages (n, %).

For comparisons across the three groups, continuous variables were analyzed using one-way analysis of variance when normality and homogeneity of variances were confirmed (via Levene’s test); otherwise, the Kruskal–Wallis H test was used when the data deviated from normality or variances were unequal. Categorical variables were compared using Pearson’s chi-squared test. Statistical significance was defined as a two-sided p-value <0.05.

Supplementary Material 6

Subgroup analysis

In the pGGN cohort, the ternary classification achieved macro-AUC of 0.7574 and 0.7965 for the internal and external validation sets, respectively (Supplementary Figure 4). For the binary classification, the model demonstrated excellent discrimination, particularly in predicting invasive adenocarcinoma for both internal and external validation sets, with macro-AUCs of 0.8738 and 0.8850, respectively. It showed moderate performance for atypical adenomatous hyperplasia, with macro-AUCs of 0.7583 and 0.7829, and the weakest performance for minimally invasive adenocarcinoma, with macro-AUCs of 0.6402 and 0.7216.

Conversely, diagnostic efficacy declined significantly in the PSN subgroup. The ternary classification yielded macro-AUCs of 0.7406 in the internal validation and 0.6485 in the external validation, with AUCs for all individual classes falling below 0.75 (Supplementary Figure 5).

Supplementary Material 7

Comparing the Performance of the Stacking Classifier, Radiomics Model, and ITH Score

Overall, 1,239 radiomics features were derived from each GGN. Subsequently, ANOVA was conducted at a significant level of $\alpha = 0.05$, engendering the retention of 954 discriminative features. To further refine this selection, recursive feature elimination with cross-validation reduced the feature set to 139 nonredundant radiomics features. Finally, radiomic features were identified using the least absolute shrinkage and selection operator method, which identified 70 features with nonzero coefficients.

The radiomics model achieved macro-AUCs of 0.6515 and 0.6161 for the internal test and external validation sets, respectively. In contrast, the ITH score demonstrated a better performance, reaching a macro-AUC of 0.7435 in the internal test set and 0.7116 in the external validation set. However, the stacking classifier outperformed both models, achieving a macro-AUC of 0.7850 for the internal test set and 0.7717 for the external validation set.

Supplementary Figure 1. Enrollment of patients with lung adenocarcinomas presenting ground-glass nodules.

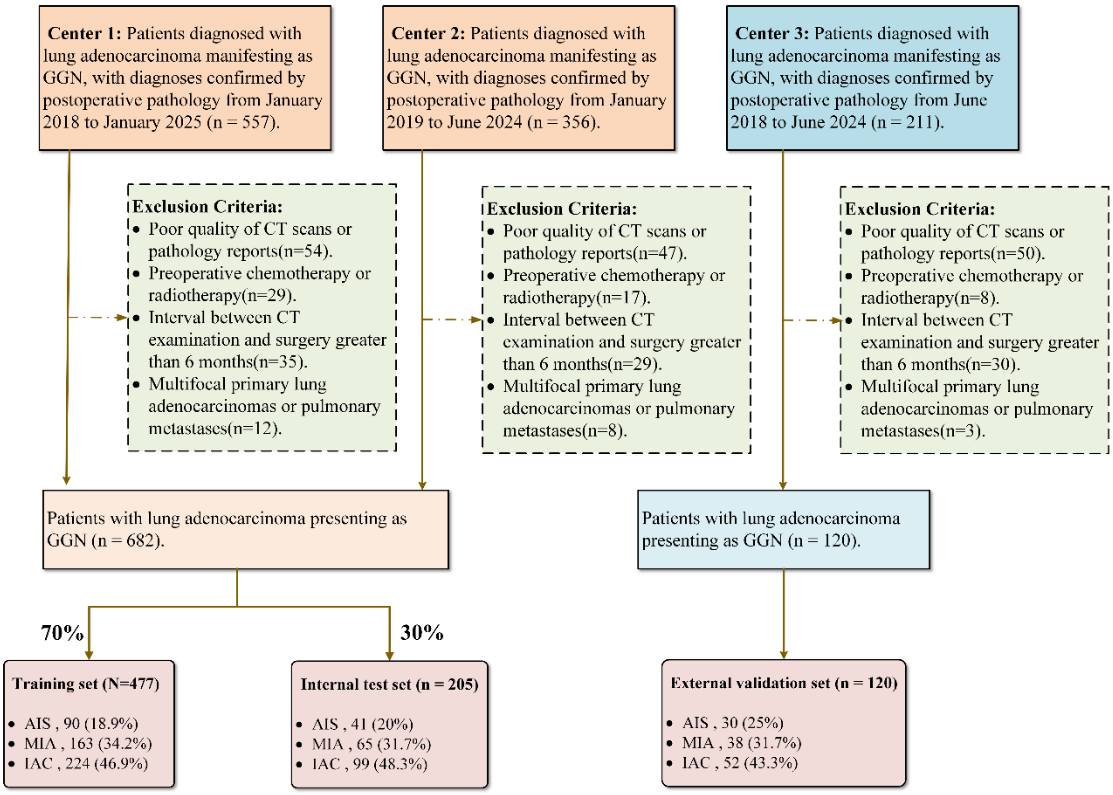

Supplementary Figure 2. Representative visualizations of the intratumoral heterogeneity scores for postoperative diagnoses of adenocarcinomas in situ, minimally invasive adenocarcinoma, and invasive adenocarcinoma.

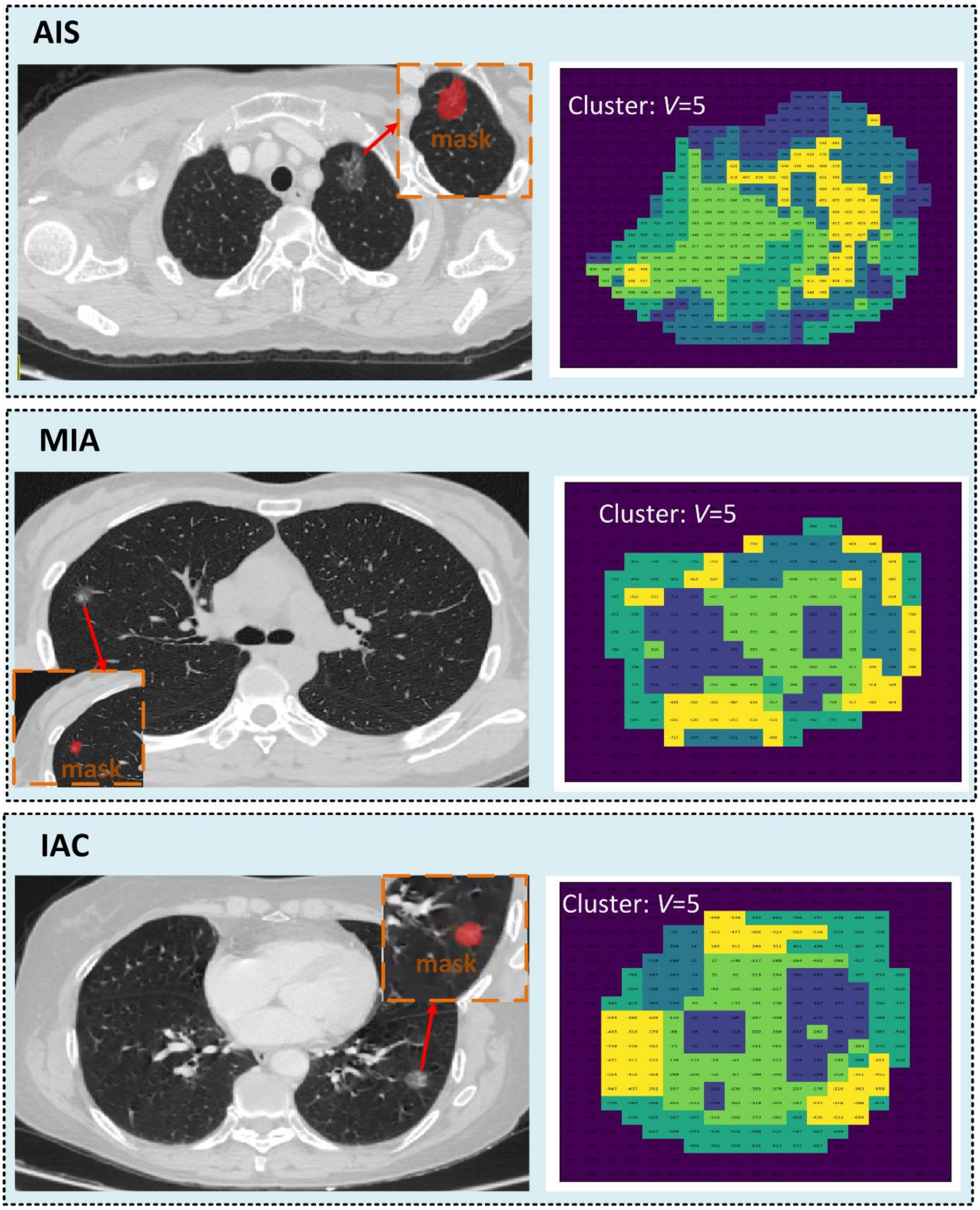

Supplementary Figure 3. Heat map of the confusion matrices for the ternary classification model (adenocarcinomas in situ vs. minimally invasive adenocarcinoma vs. invasive adenocarcinoma).

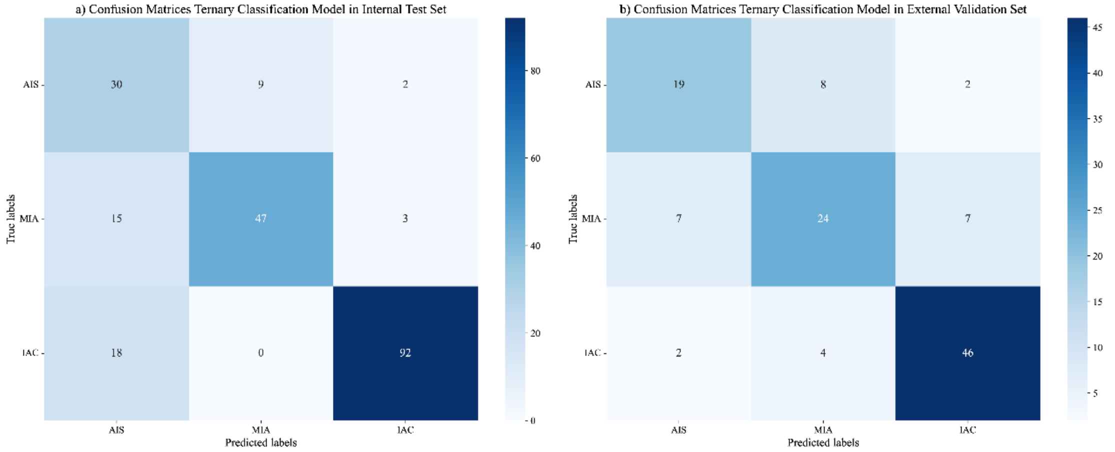

Supplementary Figure 4. Subgroup analysis of the diagnostic performance of the stacking classifier for pure ground-glass nodules. (a) Receiver operating characteristic curve for ternary classification in the internal test and (b) external validation sets. Confusion matrix for binary classification in the internal test (c) and external validation (d)sets.

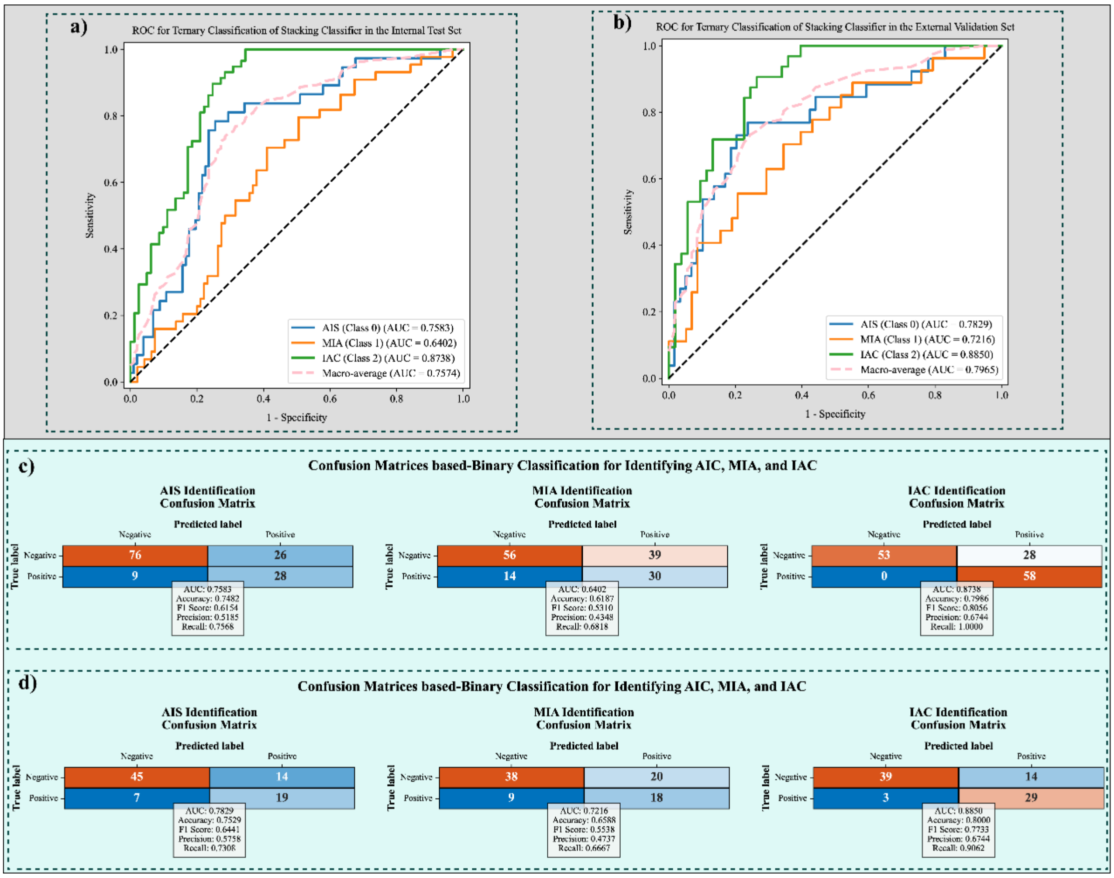

Supplementary Figure 5. Subgroup analysis of the diagnostic performance of the stacking classifier for part-solid nodules. (a) Receiver operating characteristic curve for ternary classification in the internal test and (b) external validation sets. Confusion matrix for binary classification in the internal test(c) and external validation(d) sets.

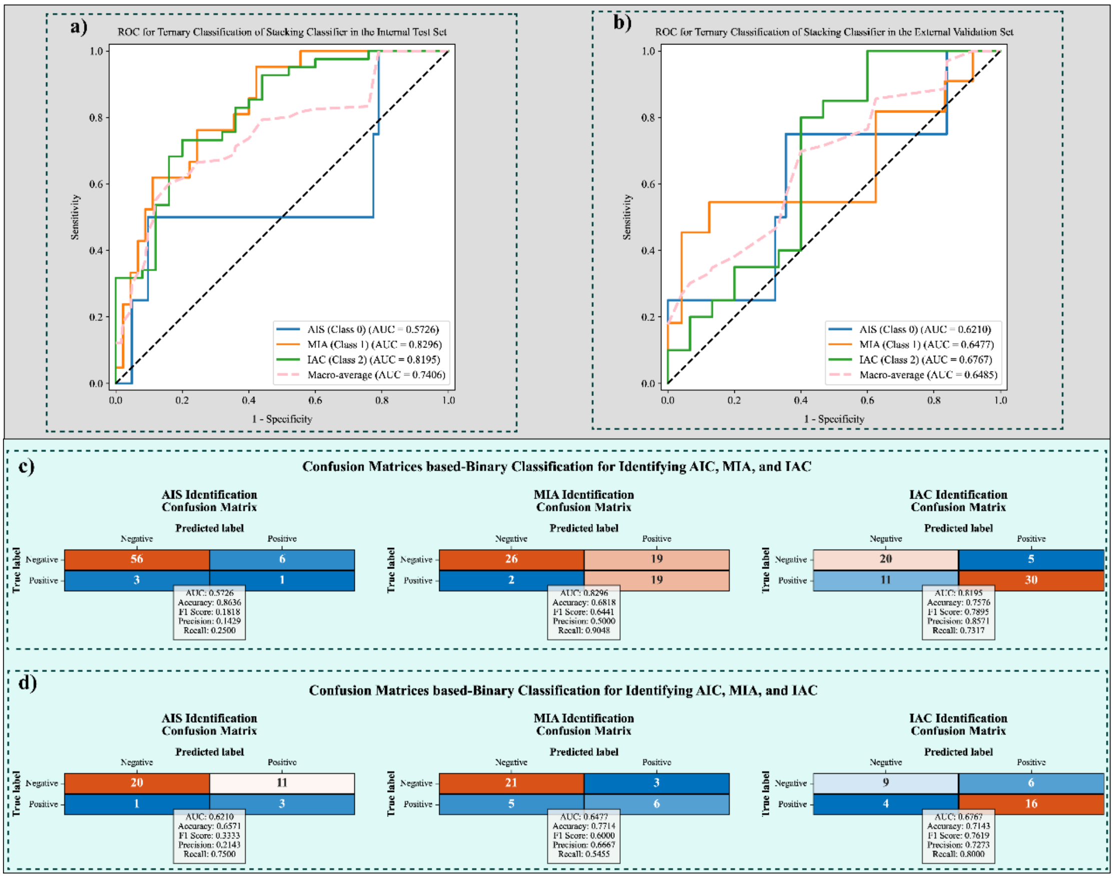

Supplementary Figure 6. Workflow of radiomics features selection and dimensionality reduction.

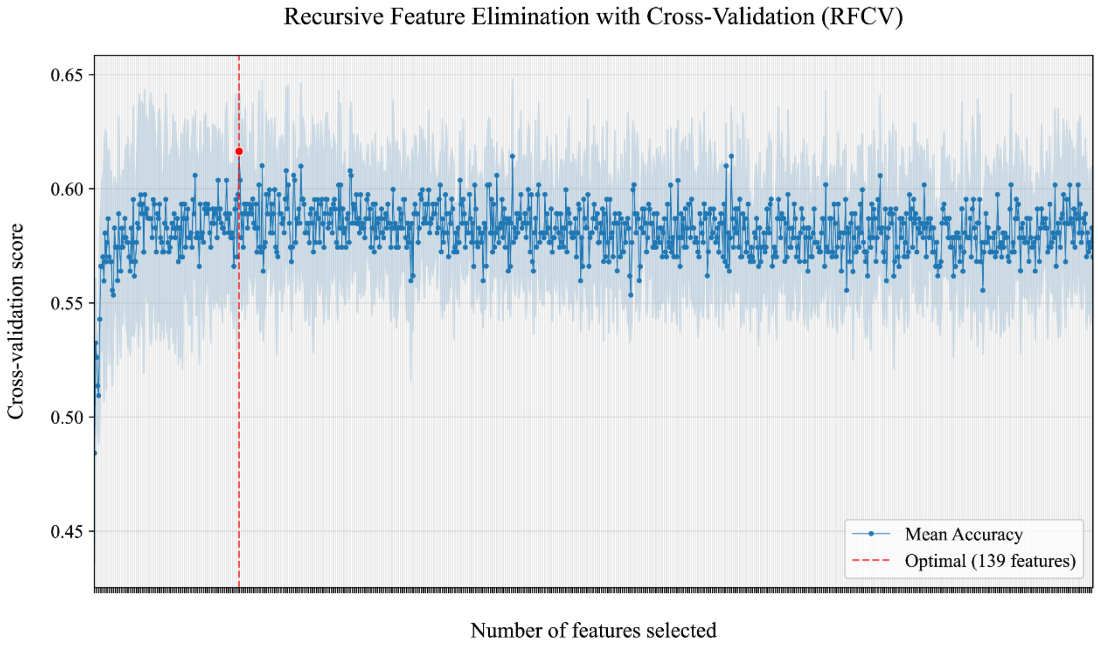

Supplementary Figure 7. Radar plots illustrating the performance of the stacking classifier, radiomics model, and intratumoral heterogeneity score in predicting outcomes within the internal test (a) and external validation (b) sets.

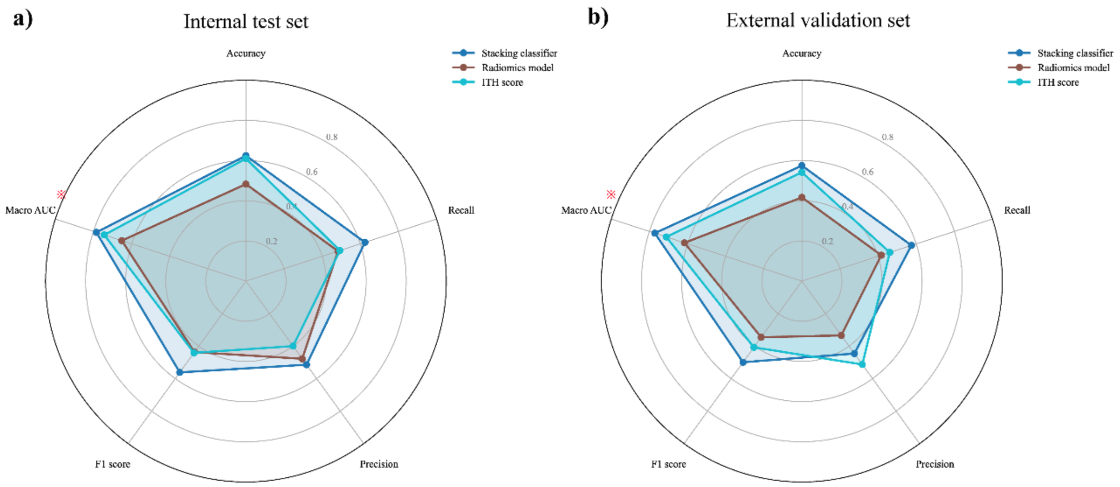

Supplementary Table 1. Comparative Analysis of Clinical-Radiological Features Among AIS, MIA, and IAC in the Training Set

Variables	AIS(N=90)	MIA(N=163)	IAC(N=224)	Total (N=477)	P value
Nodule type, n (%)					<0.001
pGGN	81 (90.00%)	120 (73.62%)	113 (50.45%)	314 (65.83%)	
PSN	9 (10.00%)	43 (26.38%)	111 (49.55%)	163 (34.17%)	
Location, n (%)					
RUL	32 (35.56%)	59 (36.20%)	86 (38.39%)	177 (37.11%)	
RML	10 (11.11%)	27 (16.56%)	33 (14.73%)	70 (14.68%)	
RLL	4 (4.44%)	8 (4.91%)	12 (5.36%)	24 (5.03%)	
LUL	28 (31.11%)	50 (30.67%)	67 (29.91%)	145 (30.40%)	

LLL	16 (17.78%)	19 (11.66%)	26 (11.61%)	61 (12.79%)	
Margin, n (%)					0.003
Well-defined	76 (84.44%)	118 (72.39%)	146 (65.18%)	340 (71.28%)	
Ill-defined	14 (15.56%)	45 (27.61%)	78 (34.82%)	137 (28.72%)	
Shape, n (%)					<0.001
Regular	66 (73.33%)	111 (68.10%)	93 (41.52%)	270 (56.60%)	
Irregular	24 (26.67%)	52 (31.90%)	131 (58.48%)	207 (43.40%)	
Lobulation sign, n (%)					0.013
Absent	48 (53.33%)	98 (60.12%)	101 (45.09%)	247 (51.78%)	
Present	42 (46.67%)	65 (39.88%)	123 (54.91%)	230 (48.22%)	

**Spiculation sign, n (%)**					**<0.001**
**Absent**	**59 (65.56%)**	**120 (73.62%)**	**115 (51.34%)**	**294 (61.64%)**	
**Present**	**31 (34.44%)**	**43 (26.38%)**	**109 (48.66%)**	**183 (38.36%)**	
**Vascular convergence sign, n (%)**					**0.034**
**Absent**	**18 (20.00%)**	**43 (26.38%)**	**35 (15.62%)**	**96 (20.13%)**	
**Present**	**72 (80.00%)**	**120 (73.62%)**	**189 (84.38%)**	**381 (79.87%)**	
**Vacuole sign, n (%)**					**<0.001**
**Absent**	**84 (93.33%)**	**139 (85.28%)**	**166 (74.11%)**	**389 (81.55%)**	
**Present**	**6 (6.67%)**	**24 (14.72%)**	**58 (25.89%)**	**88 (18.45%)**	
**Pleural indentation sign, n (%)**					**<0.001**

Absent	58 (64.44%)	95 (58.28%)	76 (33.93%)	229 (48.01%)	
Present	32 (35.56%)	68 (41.72%)	148 (66.07%)	248 (51.99%)	
Sex, n (%)					0.262
Female	69 (76.67%)	109 (66.87%)	156 (69.64%)	334 (70.02%)	
Male	21 (23.33%)	54 (33.13%)	68 (30.36%)	143 (29.98%)	
Age(y), Median (Q1, Q3)	54.00 (45.00,63.00)	56.00 (47.50,64.00)	58.00 (52.00,66.00)	57.00 (50.00,65.00)	0.001
Nodule size(mm), Median (Q1, Q3)	10.44 (8.94,14.21)	13.00 (9.85,17.59)	20.17 (15.81,23.41)	15.81 (11.18,21.10)	<0.001

Abbreviation: **pGGN, pure ground-glass nodule, PSN, part-solid nodule; AIS, adenocarcinoma in situ; MIA, minimally invasive adenocarcinoma; IAC, invasive adenocarcinoma; RUL, right upper lobe; RLL, right lower lobe; RML, right middle lobe; LUL, left**

upper lobe; LLL, left lower lobe.
